# Blumgart anastomosis reduces the incidence of pancreatic fistula after pancreaticoduodenectomy: a systematic review and meta-analysis

**DOI:** 10.1038/s41598-020-74812-4

**Published:** 2020-10-21

**Authors:** Zhenlu Li, Ailin Wei, Ning Xia, Liangxia Zheng, Dujiang Yang, Jun Ye, Junjie Xiong, Weiming Hu

**Affiliations:** 1 Department of Pancreatic Surgery, West China Hospital, Sichuan University, No. 37, Guoxue Alley, Chengdu, 610041 Sichuan Province China; 2 Key Laboratory of Transplant Engineering and Immunology, West China Hospital, Sichuan University, Chengdu, China; 3 West China School of Medicine, Sichuan University, Chengdu, 610041 Sichuan Province China; 4 Outpatient Department, West China Hospital, Sichuan University, No. 37, Guoxue Alley, Chengdu, 610041 Sichuan Province China; 5 Institute of Digestive Surgery, Sichuan University, Chengdu, Sichuan Province China; 6 Department of Gastrointestinal Surgery, West China hospital, Sichuan University, No. 37, Guoxue Alley, Chengdu, 610041 Sichuan Province China; 7 Department of Hepatobiliary-Pancreatic Surgery, Chengdu Second People’s Hospital, Chengdu, 610017 Sichuan China

**Keywords:** Risk factors, Surgical oncology

## Abstract

Postoperative pancreatic fistula (POPF) is the most serious complication after pancreaticoduodenectomy (PD). Recently, Blumgart anastomosis (BA) has been found to have some advantages in terms of decreasing POPF compared with other pancreaticojejunostomy (PJ) using either the duct-to-mucosa or invagination approach. Therefore, the aim of this study was to examine the safety and effectiveness of BA versus non-Blumgart anastomosis after PD. The PubMed, EMBASE, Web of Science and the Cochrane Central Library were systematically searched for studies published from January 2000 to March 2020. One RCT and ten retrospective comparative studies were included with 2412 patients, of whom 1155 (47.9%) underwent BA and 1257 (52.1%) underwent non-Blumgart anastomosis. BA was associated with significantly lower rates of grade B/C POPF (OR 0.38, 0.22 to 0.65; *P* = 0.004) than non-Blumgart anastomosis. Additionally, in the subgroup analysis, the grade B/C POPF was also reduced in BA group than the Kakita anastomosis group. There was no significant difference regarding grade B/C POPF in terms of soft pancreatic texture between the BA and non-Blumgart anastomosis groups. In conclusion, BA after PD was associated with a decreased risk of grade B/C POPF. Therefore, BA seems to be a valuable PJ to reduce POPF comparing with non-Blumgart anastomosis.

## Introduction

Since the first pancreaticoduodenectomy (PD) was reported by Whipple and colleagues^1^ in 1935, PD has been regarded as the standard surgical procedure for patients with either benign or malignant disease of the pancreatic head and/or periampullary region. This surgical method was considered one of the most challenging and complex abdominal operations. With advances in surgical techniques and perioperative management, the mortality caused by PD decreased to less than 5% in high-volume centres, while the rate of postoperative complications remained as high as 50%, especially postoperative pancreatic fistulas (POPF) and delayed gastric emptying (DGE)^2^.

POPF, ranging from 3 to 45% in high volume centres, was considered to be one of the most serious complications after PD^3^. This complication, as defined by the International Study Group for Pancreatic Fistula (ISGPF), is divided into 2 major groups: clinically irrelevant fistula (i.e., biochemical leak) and clinically relevant pancreatic fistula requiring a change in postoperative management (i.e., grades B and C)^4^. POPF can lead to intra-abdominal abscess, sepsis and haemorrhage and to life-threatening conditions with mortality up to 40%^5^. Therefore, numerous methods have been used to decrease POPF in previous studies, including use of octreotide^6^ or fibrin sealants to pancreatic remnant^7^, occlusion of the pancreatic duct^8^, pancreatic duct stenting^9^, modification of the pancreaticojejunostomy(PJ) anastomosis (end-to-end versus end-to-side^10^, invagination versus duct-to-mucosa^11^, interrupted suture versus continuous suture^12^) and pancreaticogastrostomy (PG)^13^. However, the reconstruction technique was perhaps the most important factor to reduce POPF. Currently, definitive evidence on the optimal surgical anastomosis technique is not yet available.

PJ was commonly used in reconstruction after PD, but the incidence of POPF remained high. PJ was further divided into two main categories, namely, duct-to-mucosa or invagination (dunking)^14^. In 2000, a novel method of PJ that combined the principle of duct-to-mucosa with the transpancreatic U suture technique was first proposed by Blumgart^15^. As opposed to the other duct-to-mucosa anastomosis such as Cattell-Warren anastomosis (CWA)^16^ and Kakita anastomosis (KA)^17^, U-sutures and the horizontal mattress suture technique was used in BA. The difference was that the Blumgart technique involved placement of 3–6 transpancreatic and jejunal seromuscular U-sutures to approximate the pancreas stump and the jejunum^18,19^. The BA has been reported to decrease the rate of grade B/C POPF to 0.67–7.14%^20–22^, significantly lower than the 10–20% in other reconstruction methods. The advantage of this technique was that U suture could avoid tangential shearing force^23,24^. Previously, BA has been reported with the advantage of reducing POPF in few case series or non-comparative retrospective studies^18,20–23,25–29^. At the same time, only one RCT^30^ and some retrospective comparative studies^19,24,31–38^ have been reported for comparison between BA and other PJ. Among some comparative studies^19,31,32,34–38^, POPF was reported to be lower in the BA group; however, other studies^24,30,33^ found no difference between the two methods. Previously, a review^39^ was published that only described a comparison between BA and KA. At present, some comparative studies focusing on BA with CWA or invagination PJ have been published. Therefore, we conducted an updated systematic review and meta-analysis to compare the safety and effectiveness of BA with that of conventional PJ after PD.

## Results

### Study selection

**Figure 1:**
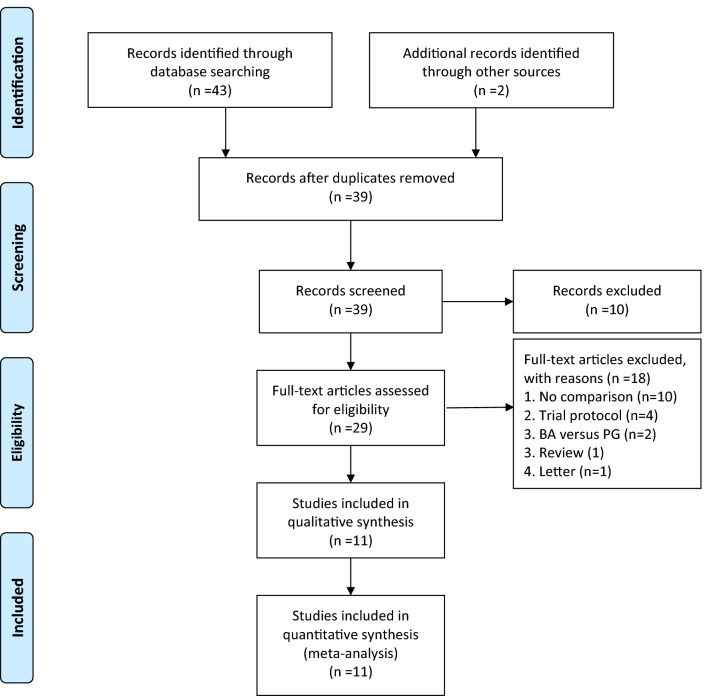
PRISMA diagram showing selection of articles for review *BA* Blumgart anastomosis, *PG* pancreaticogastrostomy.

In total, 45 studies were identified from the electronic databases, and 6 studies were excluded because they were duplicate publications. After screening the titles and abstracts, 10 records were excluded (including studies of irrelevant^40–45^, non-English^46,47^ and only abstracts^48,49^). The full texts of the remaining 29 records were assessed for eligibility. Of these, 18 were excluded because they were trial protocols^50–53^, review^39^, letter^54^, studies with no comparison with BA^18,20–23,25–29^ and studies related with BA versus pancreaticogastrostomy^55,56^. Ultimately, one RCT^30^ (from Asia) and ten non-randomized comparative studies (2 from Europe^19,38^ and 8 from Asia^24,31–37^) involving a total of 2412 patients were included in the quantitative syntheses. The process by which comparative studies were selected for inclusion in the present meta-analysis is shown in Fig. 1.

### Trial characteristics and study population

The characteristics of the included eleven studies in the meta-analysis are presented in Table 1. All studies were published between 2009 and 2019. In total, eleven studies were included with 2412 patients, of whom 1155 (47.9%) underwent BA and 1257 (52.1%) underwent non-Blumgart PJ (including 274 (21.8%) with CWA^19,24,34,38^, 672 (53.5%) with KA^31–34,37^ and 127 (10.1%) with invagination PJ^36,38^). The sample sizes ranged from 87 to 374 patients in individual studies. Four studies focused on the rate of POPF in soft pancreatic texture^30,32–34^ and eight reported the use of pancreatic duct stents, either internal or external^24,30–36^. Octreotide was used in five studies selectively^19,24,30,37,38^. Both PD and pylorus-preserving pancreaticoduodenectomy (PPPD) were reported in eleven trials and only seven studies had concomitant PV/SMV resection^24,30–32,34,35,38^. Three main methods were reported for the non-Blumgart PJ, including CWA, KA and invagination anastomosis. The ISGPF (2005) and ISGPF (2017) definitions were used in seven^19,24,31–34,37^ and four studies^30,35,36,38^, respectively. The surgical techniques and definitions of POPF are shown in Table 2.

**Table 1:** Study characteristics.

Author	Year	Country	Design	Group	No of patient	Age^#^	BMI^#^	Disease	Types of surgery	PV/SMV resection	Soft texture (%)	Stent	O	MPD (N/D)	Score*
Kleespies *et al*^19^	2009	Germany	Retro	BA CWA	92(52) 90(53)	66.5(23–82) 65 (21–78)	NA	BMDPP	PD and PPPD	NA	NA	No	S	NA	2
Fujii *et al*^31^	2014	Japan	Retro	BA KA	120(74) 120(75)	64.9(38–84) 66.0(18–83)	NA	BMDPP	PD,SSPPD and PPPD	45 40	45 45	Ex. S	NA	57/63 50/70	2
Oda *et al*^32^	2015	Japan	Retro	BA KA	78(51) 78 (50)	66 ± 10 63 ± 13	NA	BMDPP	PD	18 11	55 58	No Ex S	NA	39/39 36/42	2
Kawakatsu *et al*^33^	2018	Japan	Retro	BA KA	110(66) 176(10)	69 (21–86) 66 (32–87)	22.2(16.1–31.6) 22.3(15.9–32.0)	BMDPP	SSPPD	NA	100 100	Ex	NA	NA	2
Kojima *et al*^34^	2018	Japan	Retro	BA CWA KA	101(56) 103(55) 170(85)	71 (47–87) 68 (28–88) 70 (33–90)	NA	BMDPP	PD,SSPPD and PPPD	28 10 23	59 44 56	Ex/In Ex Ex	No	42/58 56/47 87/83	2
Lee *et al*^24^	2018	Korea	Retro	BA DtoM/CWA	43(25) 44(33)	67.00 ± 8.06 63.14 ± 10.67	24.72 ± 3.77 22.22 ± 2.91	BMDPP	PD and PPPD	5 2	NA	S	S	NA	2
Satoi *et al*^37^	2019	Japan	Retro	BA KA	118(80) 128(73)	72 (32–86) 69 (33–87)	NA	BMDPP	PD	NA	59 52	No	S	51/67 57/71	2
Hirono *et al*^28^	2019	Japan	RCT	BA CWA + KA	107(59) 103(62)	68 (24–90) 70 (40–86)	22.2(14.9–35.1) 21.6 (16.1–29.4)	BMDPP	PD and PPPD	23 24	57 56	In In	S	NA	Unclear risk
Ya-Tong Li *et al*^36^	2019	China	Retro	BA EA	201(109) 90(41)	53.28 ± 19.14 54.54 ± 17.18	NA	BMDPP	PD	NA	16 17	S	NA	NA	2
Rentao *et al*^35^	2019	China	Retro	BA EA + DtoM	148(45) 81(45)	62.5(26–86) 60 (27–74)	NA	BMDPP	PD and PPPD	2 4	48 44	In NA	NA	NA	2
Casadei *et al*^38^	2020	Italy	Retro	BA DtoM/CWA *EA*	37(22) 37(23) 37(22)	68.2 ± 10.4 68.2 ± 9.2 69.8 ± 10.5	24.9 ± 3.8 25.5 ± 3.8 24.9 ± 3.1	BMDPP	PD	0 0 0	60 49 57	NA	S	21/16 19/18 23/14	2

BMI, Body Mass Index; PV, portal vein; SMV, superior mesenteric vein; O, octreotide; MPD(N/D), main pancreatic duct(Non-dilated/dilated); BA, Blumgart anastomosis; CWA, Cattell–Warren anastomosis; KA, Kakita anastomosis; NA, Data not available; BMDPP, benign and malignant disease of the pancreatic head and the periampullary region; PD, pancreaticoduodenectomy; PPPD, pylorus-preserving pancreatoduodenectomy; SSPPD, subtotal stomach-preserving pancreatoduodenectomy; S, Select; Ex, external stent; In, internal stent; RCT, Randomized Controlled Trial; EA, Embedded anastomosis; DtoM, duct-to-mucosa anastomosis.

^#^Data was recorded as Mean ± SD or median (range).

*Randomized clinical trials (RCTs) were scored according to the RoB 2.0 of the Cochrane Collaboration; the method of McKay and colleagues was used for non-randomized studies.

**Table 2:** Surgical technique and definition of pancreatic fistula.

Author	Pancreaticojejunostomy technique	Definition of pancreatic fistula
Kleespies et al.^19^	BA: Four U-sutures, ES (DM)-PJ CWA: ES (DM)-PJ	ISGPF definition (2005)
Fujii et al.^31^	BA: ES (DM)-PJ, wide U-shape suture KA: ES (DM)-PJ	ISGPF definition (2005)
Oda et al.^32^	mBA: ES (DM)-PJ, three double-armed U-sutures KA: ES (DM)-PJ	ISGPF definition (2005)
Kawakatsu et al.^33^	mBA: ES (DM)-PJ, two or three double-armed horizontal mattress sutures and one of the sutures strode across the main pancreatic duct to bind it mKA: ES (DM)-PJ, two or three double-armed penetrating sutures	ISGPF definition (2005)
Kojima et al.^34^	mBA: three U-sutures tied at the ventral wall of the jejunum; and the use of peritoneal lavage, closed drains and dressing materials to cover the wound and drains; ES (DM)-PJ CWA: ES (DM)-PJ mKA: ES (DM)-PJ	ISGPF definition (2005)
Lee et al.^24^	BA: ES (DM)-PJ CWA: ES (DM)-PJ	ISGPF definition (2005)
Satoi et al.^37^	mBA: ES (DM)-PJ, Two U-sutures placed 0.5 cm apart from the main pancreatic duct mKA: ES (DM)-PJ, two non-absorbable interrupted penetrating sutures	ISGPF definition (2005)
Hirono et al.^30^	mBA: ES (DM)-PJ, tie knots on the ventral wall of the jejunum CWA/KA: ES (DM)-PJ, 4 trans-pancreatic sutures on KA; single layer of 8 or more sutures on CWA	ISGPF definition (2017)
Ya-Tong Li et al.^36^	BA: ES (DM)-PJ Embedded: EE (MM)-PJ	ISGPF definition (2017)
Rentao Li et al.^35^	BA/mBA: Blumgart anastomosis and its modification (superimposed the backwall sutures on each other omitted the DM anastomosis, ES (DM)-PJ In/ DtoM: EE (MM)-PJ or ES (DM)-PJ	ISGPF definition (2017)
Casadei et al.^38^	BA: ES (DM)-PJ DtoM: ES (DM)-PJ In: EE (MM)-PJ	ISGPF definition (2017)

ES, end-to-side; EE, end-to-end; DM, duct-to-mucosa; MM, mucosa-to-mucosa; IN, invagination; PJ, pancreaticojejunostomy; ISGPF, the International Study Group of Pancreatic Surgery; BA, Blumgart anastomosis; KA, Kakita anastomosis; CWA, Cattell–Warren anastomosis; DtoM, duct-to-mucosa anastomosis.

### Methodological quality of included studies

The quality assessment score of the included studies is shown in Table 1. The quality of only one RCT study was assessed using the Cochrane Collaboration Handbook^57^. The RCT trial^30^ clearly reported allocation concealment methods, withdrawals, dropouts and losses to follow-up, while not describing any kind of blinding; so, we deemed it to carry an unclear risk. The methodological quality of the included non-RCT studies was evaluated as described by McKay and colleagues^58^.

### Results of the meta-analysis and subgroup analysis

#### BA versus non-Blumgart anastomosis

##### Primary outcomes

**Figure 2:**
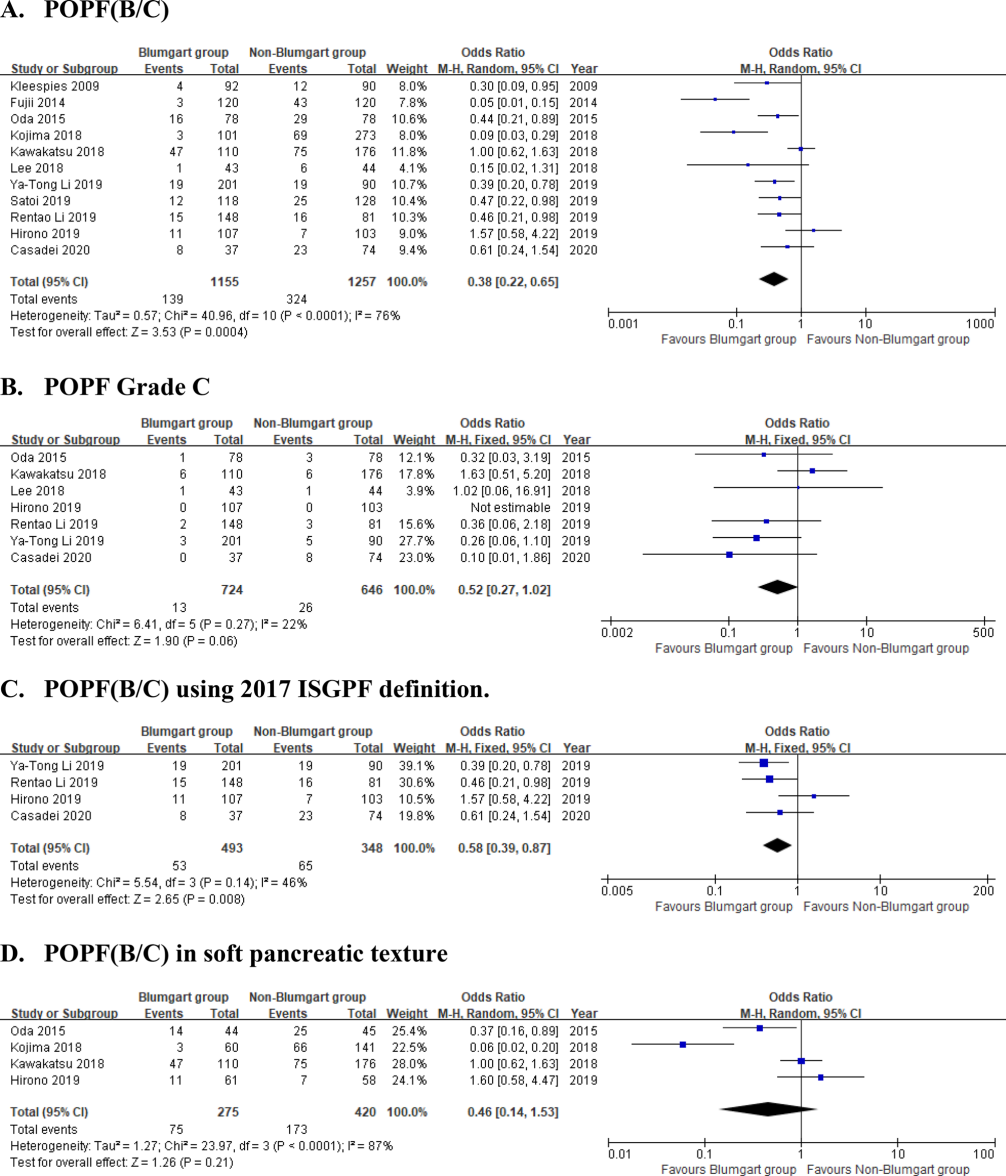
The forest plot of primary outcomes in Blumgart group versus non-Blumgart group.

The forest plots of the primary outcomes are shown in Fig. 2. All included studies reported POPF (grade B or C), while only 4 studies reported grade A or biochemical leak POPF. Therefore, we only summarized and reported the rate of grade B/C POPF. Although some degree of heterogeneity was present among these studies (*I*^2^ = 76 per cent), the use of the random-effects model did not change the result. The BA group was associated with significantly lower rates of POPF (grade B/C) (OR 0.38, 0.22 to 0.65; *P* = 0.004) and POPF (grade B/C) using 2017 ISGPF definition (OR 0.58, 0.39 to 0.87; *P* = 0.008) than non-Blumgart group. However, there was no difference in the rate of POPF (grade B/C) in soft pancreatic texture and grade C POPF between the two groups.

##### Secondary outcomes

The pooled results of the secondary outcomes of BA group versus non-Blumgart group are summarized in Table 3. In the study of Kojima^34^, conventional PJ was divided into the CWA and KA groups. The duration of the operation was significantly longer as result of the additional operation including abdominal lavage and covering the wound and drain with dressing materials; therefore, it was removed from the sensitivity analysis. In addition, the intraoperative blood loss and postoperative hospital stay were reported in the study of Kojima in the CWA and KA groups. In summary, BA were associated with significantly lower rates of overall postoperative haemorrhage (OR 0.48, 0.29 to 0.79; *P* = 0.004), intra-abdominal abscess (OR 0.53, 0.39 to 0.72; *P* < 0.0001), morbidity (OR = -0.15, -0.29 to -0.01; *P* = 0.04), and reoperation (OR 0.50, 0.30 to 0.81; *P* = 0.005) and a shorter postoperative hospital stay (Kojima-CWA group: (WMD -4.43, -7.72 to -1.15, *P* = 0.008; Kojima-KA group: (WMD -3.51, -6.35 to -0.68; *P* = 0.02). However, there were no statistically significant differences in operative time, intraoperative blood loss or other postoperative complications (DGE, bile leakage, wound infection, major morbidity and mortality) between the two groups.

**Table 3:** Results of meta-analysis comparing Blumgart group with non-Blumgart group.

Outcome of interest	Study	Patient	Effect estimate	Heterogeneity		
			OR/WMD (95%CI)	*P*	*I*^2^ (%)	*P*
**Primary outcomes**						
POPF(B/C)	11	2142	0.38 (0.22, 0.65)	*0.0004*	76	< 0.0001
Grade C pancreatic fistula	7	1370	0.52 (0.27, 1.02)	*0.06*	22	0.27
POPF(B/C) (2017 ISGPF)	4	841	0.58 (0.39, 0.87)	*0.008*	46	0.14
POPF(B/C) in soft pancreas	4	695	0.46(0.14,1.53)	*0.21*	87	< 0.0001
**Secondary outcomes**						
Operative time	9	1927	− 9.80 (− 35.81,16.20)	*0.46*	88	< 0.00001
**Blood loss**						
Kojima-CWA	10	2131	− 54.11 (− 221.63,113.42)	*0.53*	95	< 0.00001
Kojima-KA	10	2198	− 53.87 (− 220.69,112.95)	*0.53*	95	< 0.00001
Postoperative hemorrhage	8	1881	0.48(0.29,0.79)	*0.004*	0	0.44
Hemorrhage(B/C)	5	479	0.33(0.12,0.89)	*0.03*	6	0.35
DGE	4	947	0.76 (0.45,1.30)	*0.31*	76	0.006
DGE (B/C)	4	853	1.05 (0.68,1.62)	*0.83*	58	0.07
Bile leakage	6	1403	0.70(0.33,1.51)	*0.37*	0	0.63
Intra-abdominal abscess	8	1859	0.53 (0.39,0.72)	< *0.0001*	16	0.3
Wound infection	6	1358	0.65 (0.37,1.14)	*0.13*	58	0.04
Morbidity	10	2183	− 0.12 (− 0.21, − 0.04)	*0.003*	80	< 0.00001
Major morbidity	6	1518	0.67 (0.43,1.04)	*0.07*	72	0.01
Mortality	8	1599	0.51 (0.21,1.26)	*0.14*	0	0.9
Reoperation	9	1951	0.50 (0.30,0.81)	*0.005*	0	0.9
**Postoperative hospital stay**						
Kojima-CWA	11	2212	− 3.89 (− 7.45, − 0.32)	*0.03*	89	< 0.00001
Kojima-KA	11	2309	− 4.28 (− 7.35, − 1.21)	*0.006*	84	< 0.00001

WMD, Weight mean difference; POPF, postoperative pancreatic fistula; DGE, delayed gastric emptying.

#### BA versus Cattell–Warren anastomosis

##### Primary outcomes

After careful analysis, in total, four studies were related to BA versus CWA^19,24,34,38^. Detailed results are presented in Table 4 and Appendix 1. Synthesis analysis of these studies suggested that BA had significantly lower incidence of POPF (grade B/C) (OR 0.28, 0.15 to 0·52; *P* < 0.0001) than did CWA. However, there was no significant difference in grade C POPF.

**Table 4:** Results of subgroup analysis.

Outcome of interest	Study	Patient	Effect estimate	Heterogeneity		
			OR/WMD = (95%CI)	P	I^2^%	P
**Blumgart anastomosis versus Cattell–Warren anastomosis**						
**Primary outcomes**						
POPF(B/C)	4	547	0.28 (0.15, 0.52)	< 0.0001	41%	0.17
Grade C pancreatic fistula	2	161	0.19 (0.03, 1.09)	0.06	48%	0.17
**Secondary outcomes**						
Operative time	2	269	− 57.99 (− 114.22,1.76)	0.04	81%	0.02
Blood loss	3	473	− 255.09 (− 695.01, − 184.83)	0.26	89%	0.0002
Postoperative hemorrhage	3	473	0.29 (0.12,0.72)	0.008	0%	0.64
DGE	2	291	0.26 (0.10,0.68)	0.006	0%	0.9
Bile leakage	3	473	0.63 (0.21,1.88)	0.41	0%	0.54
Intra-abdominal abscess	3	473	0.53 (0.29,0.98)	0.04	2%	0.36
Wound infection	3	473	0.44 (0.09,2.21)	0.32	83%	0.003
Morbidity	2	269	0.64 (0.27, 1.54)	0.32	64%	0.09
Major morbidity	2	278	0.18 (0.01,3.38)	0.25	76%	0.04
Mortality	3	297	0.18 (0.05, 0.65)	0.009	0%	0.97
Reoperation	2	210	0.16 (0.06, 0.42)	0.0002	0%	0.9
Postoperative hospital stay	2	291	− 4.81 (− 21.66, 12.05)	0.58	93%	0.0001
**Blumgart anastomosis versus Kakita anastomosis**						
**Primary outcomes**						
POPF (B/C)	5	1199	0.26 (0.09,0.74)	0.01	89%	< 0.00001
POPF (B/C) in soft pancreas	3	531	0.30 (0.07,1.39)	0.12	90%	< 0.0001
Grade C pancreatic fistula	2	442	1.11 (0.41,2.99)	0.84	35%	0.21
**Secondary outcomes**						
Operative time	4	928	− 19.08 (− 32.11, − 6.05)	0.004	45%	0.14
Blood loss	5	1199	− 34.28 (− 62.35, − 6.02)	0.02	0%	0.63
Postoperative hemorrhage	4	959	0.58 (0.21,1.60)	0.29	27%	0.25
DGE	3	757	0.81 (0.18,3.52)	0.77	72%	0.03
Intra-abdominal abscess	3	757	0.36 (0.23,0.56)	< 0.00001	0%	0.89
Wound infection	3	757	0.44 (0.28,0.69)	0.004	69%	0.04
Morbidity	2	396	065 (032,1.35)	0.25	92%	0.0005
Major morbidity	2	517	0.72 (0.20,2.65)	0.63	89	0.003
Mortality	4	928	0.91 (0.20,4.08)	0.9	0%	0.63
Reoperation	4	928	0.68 (0.22,2.09	0.5	0%	0.89
Postoperative hospital stay	5	1199	− 6.44 (− 12.50, − 0.39)	0.04	90%	< 0.00001
**Blumgart anastomosis versus invagination pancreaticojejunostomy**						
**Primary outcomes**						
POPF (B/C)	2	365	0.43 (0.21, 0.76)	0.004	0%	0.67
Grade C pancreatic fistula	2	365	0.24 (0.06, 0.89)	0.03	0%	0.86
Severe morbidity	2	365	0.11 (0.01, 2.46)	0.17	78%	0.03
**Secondary outcomes**						
Mortality	2	365	0.38 (0.05, 3.12)	0.37	0%	0.88
Reoperation	2	365	0.41 (0.18, 0.90)	0.03	0%	0.57
Postoperative hospital stay	2	365	− 9.80 (− 15.19, − 4.14)	0.0004	80%	0.003

WMD, Weight mean difference; POPF, postoperative pancreatic fistula; DGE, Delayed gastric emptying; ICU, Intensive Care Unit; Major morbidity, Clavien-Dindo grade ≥ 3 complications.

##### Secondary outcomes

BA was associated with significantly lower rates of postoperative haemorrhage (OR 0.29, 0.12 to 0.72; *P* = 0.008), DGE (OR 0.26, 0.10 to 0.68; *P* = 0.006), intra-abdominal abscess (OR 0.53, 0.29 to 0.98; *P* = 0.04), mortality (OR 0.18, 0.05 to 0.65; *P* = 0.009), and reoperation (OR 0.16, 0.06 to 0.42; *P* = 0.0002) as well as shorter operative time (WMD -57.99, -114.22 to 1.76; *P* = 0.04) than the CWA group. There were no significant differences in other outcomes between the two groups.

#### BA versus Kakita anastomosis

##### Primary outcomes

Comparisons of BA with KA were reported in five studies^31–34,37^. Detailed results are presented in Appendix 1 and Table 4. Compared with KA, BA was associated with a significantly lower incidence of POPF (grade B/C) (OR 0.26, 0.09 to 0.74; *P* = 0.01). No significant difference was observed in POPF (grade B/C) in soft pancreas or grade C POPF.

##### Secondary outcomes

The rates of intra-abdominal abscess (OR 0.36, 0.23 to 0.56; *P* < 0.00001) and wound infection (OR 0.44, 0.28 to 0.69; *P* = 0.004) were lower in the BA group. Moreover, the BA had significantly less intraoperative blood loss (WMD − 34.28, − 62.35 to − 6.02; *P* = 0.02), shorter operative time (WMD − 19.08, − 32.11 to − 6.05; *P* = 0.004) and postoperative hospital stay (WMD − 6.44, − 12.50 to − 0.39; *P* = 0.04). There were no significant differences in other outcomes.

#### BA versus invagination PJ

Only two studies^36,38^ could be used for this issue. The results are shown in Table 4 and Appendix 1. BA was associated with significantly lower rates of POPF (grade B/C) (OR 0.43, 0.21 to 0.76; *P* = 0.004), grade C POPF (OR 0.24, 0.06 to 0.89; *P* = 0.03) and reoperation (OR 0.41, 0.18 to 0.90; *P* = 0.03), as well as shorter postoperative hospital stay (WMD − 9.80, − 15.19 to − 4.14; *P* = 0.0004) than invagination PJ. However, major morbidity and mortality were comparable between the two approaches.

### Publication bias

**Figure 3:**
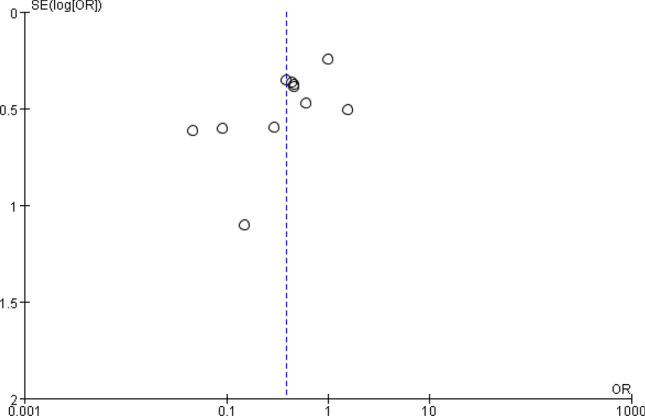
Funnel plot to investigate publication bias basing on POPF.

To examine any publication bias in the included studies, a funnel plot was constructed using the Review Manager 5.3. The funnel plot based on grade B/C POPF is shown in Fig. 3. The funnel plot was asymmetric; therefore publication bias might exist.

## Discussion

Until now, the optimal reconstruction technique for PJ after PD has remained controversial^59^. This systematic review and meta-analysis not only made a comparison between BA and non-Blumgart PJ, but it also compared BA with CWA, KA and invagination PJ. This study suggested that the rates of grade B/C POPF, morbidity and postoperative haemorrhage were significantly lower in the BA group than in the non-Blumgart group. Therefore, BA appeared to be a safe, feasible and effective PJ technique compared to non-Blumgart PJ.

According to the previous reports, there are a number of plausible explanations for why BA was superior to a non-Blumgart anastomosis procedure in reducing the POPF rate. First, BA reduces tangential tension and shear force at the pancreatic stump via the use of the transpancreatic U-sutures. Second, BA maintains the pancreatic stump with a sufficient blood supply by interrupted mattress U-sutures. Furthermore, BA guarantees a tension-free approximation between the posterior and anterior seromuscular jejunum and excellent visualization of the pancreatic duct by placing a duct-to-mucosal suture at the beginning^18,19,21,22,27,33^. However, several drawbacks have also been reported regarding BA, especially for the original BA. King et al*.*^28^ reported that BA was incomplete and resulted in an unstable covering of pancreas stump that is prone to evoke POPF when joining a thin jejunum and a thick pancreas. To further achieve improvement, accumulated modifications of Blumgart anastomosis were proposed, including utilization of one suture for the anterior and posterior wall^19^, knot-tying on the ventral wall of the jejunum^28,30^, the use of closed drains and dressing materials to cover the wound and drains^34^, and a wide U-shape suture^31^ that minimized the space between the knots. Recently, Hirono et al*.*^30^ suggested that pancreatico-enteric anastomosis should use as few sutures as possible, taking care to not tie the suture too tightly and thus maintaining blood flow in the pancreatic stump.

The definition and classification of ISGPF was used in all the included studies. However, the ISGPF was updated in several studies, and the POPF grade A was called a “biochemical leak” because it has no significance in clinical practice. However, the definitions of grade B/C POPF are not very different between the 2005 and 2017 ISGPF. In addition, all included studies reported grade B or C POPF, while only 4 studies reported all POPF (including grade A or biochemical leak, grade B and grade C). Therefore, in the analysis of postoperative outcomes following PD, the present study mainly focused on grade B/C POPF^60^. In the present meta-analysis, the BA group had a lower rate of grade B/C POPF (8.3% vs 22.4%, *P* = 0.0004) than the non-Blumgart group, which was similar to the result of a previous study^39^_._ The incidences of grade B/C POPF after BA ranged from 0 to 30.8% as has been described in previous case series studies (Table 5). One of the important factors that affected the development of POPF was pancreatic texture. For soft pancreatic texture, the incidence of POPF (grade B/C) was lower in the BA group than in the conventional PJ group (27.3% versus 41.2%), although there was no statistically significant difference (OR 0.46, 0.14 to 1.53; *P* = 0.21).Therefore, it is possible that a soft pancreas led to a high incidence of pancreatic fistula, regardless of which way the PJ anastomosis was used.

**Table 5:** Summary of excluded literature reports for Blumgart Anastomosis.

Study	Year	Country	Group	No of patient	Age	Soft texture (%)	CR-POPF (%)	PPH (%)	Morbidity (%)	Mortality (%)	POHS
Grobmyer et al^20^	2010	USA	BA	187	63 (23–85)	47	6.9	3.2	1.6	1.6	10 (7–58)
Mishra et al^18^	2011	India	BA	98	48.6 (16–76)	43.9	7.14	5.1	39.8	3.06	13 (6–41)
Kim et al^55^	2014	Korea	BA	20	63.5 ± 9.7	NA	10	10.0	20.0	0	21.5 ± 7.0
Wang et al^56^	2016	China	BA	103	65 (30–87)	NA	20	12	49.0	0	25 (10–99)
Poves et al^25^	2017	Spain	OBA	13	67 ± 10.5	76.9	30.8	15.4	84.6	0	21 (13.5–42.5)
			LBA	13	65 ± 11.8	76.9	15.4	7.7	69.2	0	14 (7.5–15.5)
Lee WJ et al^21^	2018	Korea	BA	11	NA	NA	0	NA	NA	0	NA
Wang et al.^26^	2018	China	OBA	87	NA	55.2	8.0	3.4	43.7	NA	24 (7–77)
			RBA			52.9	12.6	13.8	37.9		24 (9–136)
Wang et al^27^	2018	China	cBA	97	NA	40.2	10.3	0	55.5	1.0	22 ± 10
			mBA	50		50.0	12.0	0	40	0	23 ± 8
Gupta et al^23^	2019	India	BA	81	48.04 ± 10.14	45.7	12.3	13.6	51.9	NA	15 (7–65)
Kim et al^28^	2019	Korea	mBA	50	67.2 ± 3.6	24.0	10	4.0	10.0	2.0	19.5 ± 2.6
Tewari et al^22^	2019	India	BA	150	51.2 ± 10	74.6	0.67	NA	NA	0	7.3 ± 4.2
Nagakawa et al^29^	2020	Japan	LBA	20	62.1 ± 16.8	95.0	20.0	NA	25.0	NA	23.9 ± 15.6
			LBA with clips	19	60.4 ± 17.3	100	21.1		26.3		22.1 ± 12.1

Data was recorded as Mean ± SD or median (range).

BA, Blumgart anastomosis; OBA, open pancreaticoduodenectomy with Blumgart anastomosis; LBA, laparoscopic pancreaticoduodenectomy with Blumgart anastomosis; RBA, robotic pancreaticoduodenectomy with Blumgart anastomosis; cBA, conventional Blumgart anastomosis; mBA, modified Blumgart anastomosis; POHS, postoperative hospital stay; NA, Data not available, CR-POPF, clinically relevant postoperative pancreatic fistula; PPH, postoperative hemorrhage.

Previous studies have suggested that POPF was the main cause for intra-abdominal abscess, postoperative haemorrhage and DGE after PD^2^. Thus, to some extent, it is clear that once the incidence of POPF decreases, perhaps postoperative morbidity would significantly decline. Our analyses indicated that the rates of intra-abdominal abscess and postoperative haemorrhage were significantly lower in the BA group (9.1% vs 16.5%, *P* < 0.0001), which was mainly due to the absence of dead space between the pancreatic cut surface and the jejunal wall in the U-suture technique group^30^. According to the results of the current meta-analysis, BA might significantly minimize the rate of reoperation (3.0% vs 4.9%, *p* = 0.005). The incidence of reoperation mainly resulted from severe complications including POPF (grade B/C), bleeding, and abscess formation. Therefore, the rate of overall postoperative morbidity and mortality in the BA group were 23.7% and 0.9%, respectively, less than in previous studies. At the same time, because of the decrease in complications, postoperative hospital stays were also reduced. The subgroup analysis that focused specifically on clinical trials comparing Blumgart anastomosis with other types of PJ anastomosis still favoured the advantages of BA.

There were some limitations in our meta-analysis that should be acknowledged. First, most included studies were retrospective before–after studies that inevitably led to selection bias. Second, the Blumgart technique was slightly different among studies with several modifications. Third, there was probably publication bias in the current study, mainly due to the unpublished studies with negative results.

### Conclusions

In conclusion, compared with non-Blumgart PJ, BA was safer and more effective after PD with a lower incidence of grade B/C POPF, comparable operative time and intraoperative blood loss, lower morbidity and a shorter postoperative hospital stay. However, before recommending widespread use, it is necessary to design prospective multicenter, high quality RCTs to further test and verify the advantages of BA in patients with soft pancreas.

## Materials and methods

### Study design

The review was established according to the Preferred Reporting Items for Systematic review and Meta-Analysis (PRISMA) guidelines^61^. Two researchers (ZLL and ALW) independently conducted a comprehensive and systematic search of PubMed, EMBASE, Web of Science and the Cochrane Central Library from January 2000 (the first Blumgart anastomosis was described in 2000) to March 2020. The following search terms were chosen to screen databases, such as pancreaticoduodenectomy, pancreatoduodenectomy, Whipple, Blumgart, pancreaticojejunostomy, duct-to-mucosa and invagination along with their synonyms or abbreviations. The complete retrieval strategy in PubMed as follows:

- #1 Pancreaticoduodenectomy [Mesh]
- #2 Pancreaticoduodenectom*[tw] OR Pancreatoduodenectom*[tw] OR Duodenopancreatectom*[tw] OR Duodenum [tw] OR Pancreatectomy [tw] OR Whipple [tw]
- #3 #1 OR #2
- #4 Blumgart [tw]
- #5 Pancreaticojejunostomy [Mesh]
- #6 Pancreaticojejunostom*[tw] OR Pancreatojejunostom*[tw] OR duct-to-mucosa [tw] OR invagination [tw]
- #7 #5 OR #6
- #8 "2000/01/01"[dp]: "2020/03/31"[dp]
- #9 #3 AND #4 AND #7 AND #8

Relevant papers have also been identified from the bibliographies of papers.

### Inclusion and exclusion criteria

The studies were included based on the following criteria: English language articles published in peer-reviewed journals; human studies; studies with at least the primary outcome mentioned; only comparative clinical trials with full-text descriptions; clear documentation of the PJ technique and where multiple studies came from the same institute and/or authors, either the higher quality study or the more recent publication was included in the analysis. The following studies were excluded: abstracts, letters, editorials, expert opinions, case reports, reviews, trial protocols, and studies related to comparing BA with PG.

### Outcomes of interests

Perioperative outcomes and postoperative complications were evaluated. The primary outcome measure was postoperative pancreatic fistula (POPF). The POPF was defined according to the 2005^62^ or 2017^4^ International Study Group of Pancreatic Fistula (ISGPF) criteria. POPF (grade B/C) was a combination of grade B and C and was associated with a clinically relevant condition related directly to POPF. Secondary outcome included postoperative complications (postoperative haemorrhage, DGE, postoperative intra-abdominal abscess, wound infection, morbidity, mortality, reoperation) and perioperative outcomes (operative time, intraoperative blood loss, postoperative hospital stay). Bile leakage was defined as any biliary output via percutaneous drains after the first postoperative day, or detected at a reoperation. DGE and postoperative haemorrhage were defined and graded in accordance with the 2007 ISGPS criteria^63,64^. Postoperative morbidity was defined as total complications from date of operation to discharge. According to the modified Clavien-Dindo classification^63–65^, the Clavien-Dindo grade ≥ 3 complications were regarded as major morbidity. Mortality was defined as the number of deaths from any cause occurring in hospital or within 30 days after operation. Reoperation was defined as the need for laparotomy as a consequence of the first operation.

### Data extraction and quality assessment

Data were extracted independently by two reviewers using standard forms and were cross-checked. Inconsistencies were resolved through discussion until consensus was reached, or a third reviewer would take part in the discussion. The RCT was assessed according to the Cochrane Collaboration Handbook^57^. The scoring system included the following criteria: random sequence generation, allocation concealment, blinding of participants and personnel, blinding of the results assessment, incomplete data of the results, selective reporting, and other sources of bias. Observational studies were assessed as described by McKay and colleagues^58^, including assessment of data collection (prospective versus retrospective), assignment to BA or non-Blumgart PJ group by means other than the surgeon’s preference, and an explicit definition of POPF (studies were given a score of 1 for each of these areas, giving a total score of 1–4). Continuous variables were presented as the mean with corresponding standard deviations to be pooled in the meta-analysis. When the trials had reported medians and ranges instead of means and standard deviations, the estimation methods were used basing on the literature^66,67^.

Quantitative data was extracted from the selected studies, including population characteristics (age, gender, BMI), intraoperative conditions (type of anastomosis, pancreatic texture, mean main pancreatic diameter, operative time and intraoperative blood loss) and postoperative parameters (POPF(grade B/C), DGE, intra-abdominal abscess, bile leakage, wound infection, morbidity, mortality, reoperation, duration of drainage and postoperative hospital stay) in each study.

### Statistical analysis

Data analyses were performed using Review Manager 5 software (The Cochrane Collaboration, Oxford, UK). Heterogeneity was evaluated by means of the *χ2* test, with *P* ≤ 0.10 considered to represent a significant difference. *I*^2^ values were used for the evaluation of statistical heterogeneity; an *I*^2^ value of 50% or more indicated the presence of heterogeneity^68^. Initially, a fixed-effects model was used to synthesize all data. With regard to outcomes when significant heterogeneity existed across studies, sensitivity analysis was performed by sequentially omitting each study to test the influence of an individual study on pooled data. However, if there was evidence of heterogeneity among the included studies, random-effects analysis according to DerSimonian and Laird^69^ was used. Clinical heterogeneity could be explained by different definitions of outcome parameters, and variability of interventions and perioperative management. The result of meta-analysis was presented as WMD or OR with 95%confidence intervals (CI). Data analysis was performed by comparing BA versus non-Blumgart PJ (including CWA, KA and invagination PJ). Funnel plots were constructed to evaluate potential publication bias, based on the grade B/C POPF.

## Supplementary information

Supplementary Information 1.
