# Supplementary material for: Blumgart anastomosis reduces the incidence of pancreatic fistula after pancreaticoduodenectomy: a systematic review and meta-analysis
Source: Sci Rep. 2020 Oct 21;10:17896. doi: 10.1038/s41598-020-74812-4 (PMC7578105; doi:10.1038/s41598-020-74812-4)
Supplement: Supplementary file 1 — Supplementary Information 1. [file 41598_2020_74812_MOESM1_ESM.docx]

**Blumgart Anastomosis Reduces the Incidence of Pancreatic Fistula after Pancreaticoduodenectomy. A Systematic Review and Meta-analysis.**

Zhenlu Li^1, 3 +^, Ailin Wei^1, 2 +^, Ning Xia^3^, Liangxia Zheng^4^, Dujiang Yang^3, 5, 6^, Jun Ye^7^, Junjie Xiong^1, *^, Weiming Hu^1,^ ^*^

**A**


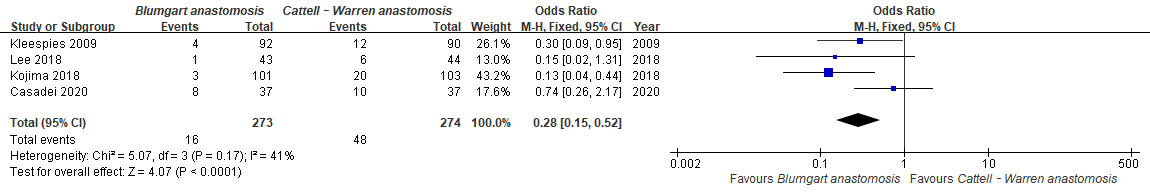


**B**
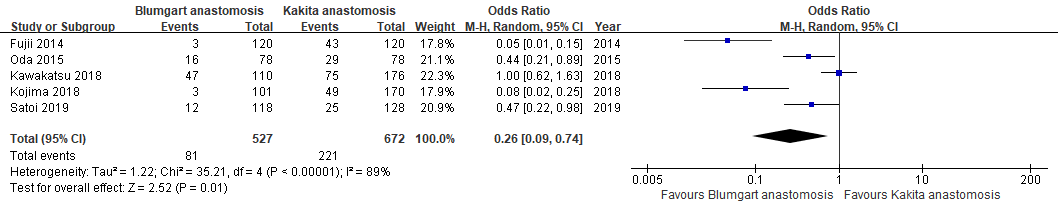


**C**


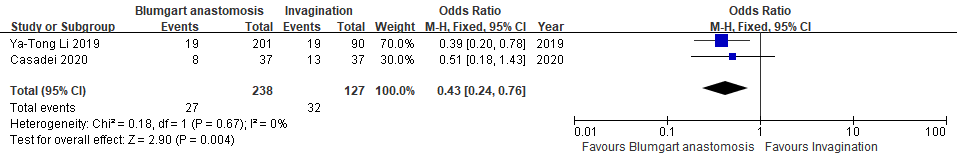


**Appendix 1** The forest plot of postoperative pancreatic fistula (grade B or C) in subgroups.

(A) Blumgart anastomosis vs Cattell-Warren anastomosis;

(B) Blumgart anastomosis vs Kakita anastomosis;

(C) Blumgart anastomosis versus invagination pancreaticojejunostomy.
